# Less meat in the shopping basket. The effect on meat purchases of higher prices, an information nudge and the combination: a randomised controlled trial

**DOI:** 10.1186/s12889-022-13535-9

**Published:** 2022-06-07

**Authors:** R. E. Vellinga, M. Eykelenboom, M. R. Olthof, I. H. M. Steenhuis, R. de Jonge, E. H. M. Temme

**Affiliations:** 1 National Institute for Public Health and the Environment, Antonie van Leeuwenhoeklaan 9, Bilthoven, 3721 MA The Netherlands; 2 Department of Health Sciences, Faculty of Science, Vrije Universiteit Amsterdam, Amsterdam Public Health Research Institute, De Boelelaan 1085, 1081 HV Amsterdam, the Netherlands

**Keywords:** Meat tax, Randomised controlled trial, Fiscal policy, Fiscal measure, Food policy measures, Information nudge

## Abstract

**Background:**

Reduced meat consumption benefits human and planetary health. Modelling studies have demonstrated the significant health and environmental gains that could be achieved through fiscal measures targeting meat. Adding other interventions may enhance the effect of a fiscal measure. The current study aimed to examine the effect of higher meat prices, an information nudge and a combination of both measures on meat purchases in a three-dimensional virtual supermarket.

**Methods:**

A parallel designed randomised controlled trial with four conditions was performed. Participants (≥ 18 years) were randomly assigned to the control condition or one of the experimental conditions: a 30% price increase for meat (‘Price condition’), an information nudge about the environmental impact of meat production and consumers’ role in that regard (‘Information nudge condition’) or a combination of both (‘Combination condition’). Participants were asked to shop for their household for one week. The primary outcome was the difference in the total amount of meat purchased in grams per household per week.

**Results:**

Between 22 June 2020 and 28 August 2020, participants were recruited and randomly assigned to the control and experimental conditions. The final sample included 533 participants. In the ‘Combination condition’, − 386 g (95% CI: − 579, − 193) meat was purchased compared with the ‘Control condition’. Compared to the ‘Control condition’ less meat was purchased in the ‘Price condition’ (− 144 g (95%CI: − 331, 43)), although not statistically significant, whereas a similar amount of meat was purchased in the ‘Information nudge condition’ (1 g (95%CI: − 188, 189)).

**Conclusion:**

Achieving the most pronounced effects on reduced meat purchases will require a policy mixture of pricing and informational nudging. Less meat is purchased in a virtual supermarket after raising the meat price by 30% combined with an information nudge. The results could be used to design evidence-based policy measures to reduce meat purchases.

**Trial registration:**

The trial was registered in the Netherlands Trial Register identifier NL8628. Registered on 18/05/2020. ICTRP Search Portal (who.int) NTR (trialregister.nl).

**Supplementary Information:**

The online version contains supplementary material available at 10.1186/s12889-022-13535-9.

## Introduction

A large body of evidence shows that the production and consumption of meat are associated with climate change, loss of biodiversity, occupation of large areas of land and alterations of the nitrogen cycle, and contribute to acidification and eutrophication [1]. Moreover, high red and processed meat consumption, with 100–120 g and 50 g respectively, are associated with a 10–20% greater likelihood of developing cancer, diabetes, stroke, coronary heart disease and heart failure, and substantially contribute to the foodborne burden of disease [2, 3]. Current global consumption levels of red and processed meat exceed the recommendations [4]. A lower consumption of meat could significantly reduce the effects of food consumption on the environment, improve human health and lead to a net societal benefit [5].

Multiple types of food policy interventions (e.g. informative, administrative, behaviour and market-based instruments) can be implemented to steer consumers’ dietary choices [6]. A scoping review showed that hardly any currently implemented food policy worldwide focuses specifically on the reduction of meat consumption [6]. Most implemented food policies focus on public health and lower consumption of energy-dense, (micro)nutrient-poor foods and beverages, and higher vegetable and fruit consumption [6]. Policies combining health and sustainability objectives are few and often only implemented via informative measures [6]. Such measures from a freedom-of-choice perspective include, for example, dietary guidelines that recommend a maximum consumption of meat. However, their impact is low, not assessed or difficult to measure [7].

Modelling studies have demonstrated the significant health and environmental gains that could be achieved by lower meat consumption through higher meat prices [5, 8, 9]. A recent social cost and benefit analysis (SCBA) from the Netherlands estimated over a period of 30 years that the average meat consumption decreased by 16% from 107 to 90·3 g per person per day after a 30% price increase on meat [5]. Adding other measures, such as information or nudges to create awareness among consumers, may enhance the effect of a fiscal measure since mixes of instruments are often more effective compared with one specific instrument only [7, 10]. Information nudges alter consumer behaviour from a freedom-of-choice perspective to a more healthy choice and may contribute to improving population dietary behaviours [11].

Food prices are known to be a significant driver for food choices [12]. Systematic reviews show that taxing unhealthy foods to discourage their consumption is an effective measure to improve dietary behaviour [13, 14]. For example, in a systematic review and meta-analysis, Teng et al. (2020) demonstrated with real-life evaluations that the equivalent of a 10% tax on sugar-sweetened beverages was associated with an average decline in beverage purchases and dietary intake of 10% [15]. Fiscal measures such as taxation could be a powerful measure targeting meat reduction as higher prices discourage consumers from purchasing the foods that are taxed [13].

However, limited empirical evidence is available on the effectiveness of higher meat prices as no fiscal policies that aim to reduce meat purchases or consumption have been implemented as yet. Although more literature has become available on small-scale experiments that target meat consumption, these experiments are mostly focused on changing attitudes and intention or willingness to consume meat and not on actual purchases [16, 17]. One small-scale experiment did investigate the effect on purchases of altering prices. Garnett et al. (2021) studied the impact on sales of experimentally altering the price of meat and vegetarian meal options in a college cafeteria in the UK [18]. The price differentiation increased the sales of vegetarian meals but did not affect the sales of meat meals.

Robust evidence on policy measures to decrease meat purchases and consumption is needed, as effective evidence-based interventions are still lacking, and evidence currently relies on modelling studies. Therefore, this study presents a randomised controlled trial (RCT) which examined the effect of a fiscal measure (higher meat prices), an information nudge (information on the environmental impact of meat production and the role of the consumer in that regard) and a combination (higher meat prices and an information nudge) on meat purchases in a Dutch virtual supermarket.

## Methods

### Study design

A parallel designed RCT with four conditions was conducted. The trial was registered in the Netherlands Trial Register identifier NL8628. Registered on 18/05/2020. Participants were randomised to one of the following conditions:

(i) An experimental condition ‘Price condition’: prices of meat and meat products (containing at least 80% meat) were increased by 30% at the consumer food purchase level. A price increase of 30% was chosen because it was previously estimated that such a price increase could lead to a net societal benefit for the Netherlands [5]. Forty-four meat products were taxed (Supplemental Table 1). The average price of meat increased from €2·87 to €3·73 per unit as sold. No information was given on the purpose of the revenue. In order to reflect a real-world setting, participants were made aware of the price increase of meat via a notification before entering the supermarket: “The government has increased the tax on meat in the virtual supermarket, leading to a price increase of 30% for meat”.
(ii) An experimental condition ‘Information nudge condition’: participants were exposed to an information nudge, as framed within the typology of interventions in proximal physical micro-environments [19]. The nudge aimed to create awareness regarding the environmental impact associated with meat production and to influence the consumer’s role in that regard. Before entering the virtual supermarket, participants were exposed to the information nudge: “The government wants to reduce the consumption of meat in the Netherlands because meat production damages the environment. You can help to reduce the environmental damage caused by meat production by purchasing less meat”. Regular food prices were used.
(iii) An experimental condition ‘Combination condition’: both higher prices (30% price increase on meat, condition i) and the information nudge (condition ii) were included. Participants were exposed to the notification and nudge before entering the virtual supermarket: “The government wants to reduce the consumption of meat in the Netherlands because meat production damages the environment. You can help to reduce the environmental damage caused by meat production by purchasing less meat. The government has increased the tax on meat in the virtual supermarket, leading to a price increase of 30% for meat”.
(iv) A control condition ‘Control condition’: regular food prices were used, and participants did not receive a notification before entering the virtual supermarket.

### The virtual supermarket

The study was conducted in a Dutch virtual supermarket, which is a three-dimensional computer software system simulating the in-store environment of a real supermarket [20]. The tool enables participants to purchase food items and measures food purchasing behaviour in a virtual setting. A validation study, where shopping patterns in the virtual supermarket were compared with those in real life, found that the software is a valid tool for measuring food purchasing behaviour in a supermarket setting [21]. The software was updated in 2019 and is described in detail elsewhere [22]. The updated version includes new functionalities and features to create a more realistic virtual supermarket. The virtual supermarket contained 580 foods and proportionally represented the usual supermarket offer. The selection of available foods was based on the stock of the leading supermarket chain and supplemented with the most frequently consumed foods within the most recent Dutch National Food Consumption Survey 2012–2016 [23]. The leading supermarket chain’s website was assessed in February 2020 and provided information on product weight and food prices. Foods were coded according to the Dutch Food Composition Database (NEVO) (NEVO online version 2019/6.0) [24].

### Participants

Eligible participants were adults (≥ 18 years) with an adequate command of the Dutch language, largely or totally responsible for grocery shopping for the household and with access to a laptop or computer. Participants were recruited via an online research panel in the Netherlands (Panel Inzicht) and were rewarded with virtual points which could be redeemed for cash. The study purposes were not mentioned during recruitment nor study execution. Participants for the current study were recruited simultaneously with participants for another project which aimed to evaluate the effects of a sugar-sweetened beverage tax and a nutrient profiling tax on consumer purchases (Netherlands Trial Register registration number NL8616) [22]. This other project determined the total number of included participants per study condition (*n* = 109). Data from participants who were exposed to the control condition were used in both studies for the control condition. The Research Ethics Review Committee of the Faculty of Sciences, Vrije Universiteit Amsterdam gave its ethical agreement (reference 20,205). Approval from the Dutch Medical Research Involving Human Subjects Act further was not needed.

### Procedure

The full procedure of this study is described elsewhere [22]. In short, after participants were recruited and completed the screening questionnaire, they were invited via email to participate in the study. Eligible participants who provided informed consent were sent instructions to download and install the virtual supermarket software. Participants were randomly sent log-in codes in order to be assigned to one of the conditions. After logging in, participants were asked about their household size and composition in order to determine their weekly shopping budget. The National Institute for Family Finance Information (NIBUD) provided standardised household budgets [25]. Instructions were given to do a weekly grocery shop in the supermarket for their household (seven times breakfast, lunch, dinner and snacks). It was explained that participants would not receive the purchased groceries nor budget in real life. Before entering the virtual supermarket, participants in the experimental conditions were exposed to the notifications corresponding to the conditions. After the check-out in the supermarket, participants were directed to a final questionnaire covering various questions about the experiment and participant characteristics. The entire study was executed online.

### Outcomes

The difference in the total amount of meat purchased (in g) per household per week was the primary outcome. Information on the purchases was collected for meat purchased (binary: yes/no), meat and total food items purchased (in n), the environmental impact and nutritional outcomes. The environmental impact of foods was derived from the Dutch Life Cycle Assessment (LCA) Food database [26]. The LCA’s quantify the environmental impact of a food from cradle to plate and are described in more detail elsewhere [27]. Indicators included were greenhouse gas (GHG) emissions (in kg CO_2_-equivalents), blue water consumption (water sources from surface or groundwater resources) (in m^3^) and land use (in m^2^/year). GHG emissions is used as a proxy for other indicators as GHG emissions highly correlates with other environmental indicators. Blue water consumption and land use had the weakest correlation with GHG emissions in the Dutch LCA Food database and are therefore also included in this study [27]. The LCA’s were linked via NEVO codes. For which no primary LCA data were available the LCA’s were linked to similar foods based on similarities in types of food, production systems and ingredient composition. For composite dishes, standardized recipes from the Dutch food composition database (NEVO-online version 2016/5.0) were used where available and if not available, recipes were based on label information [24]. The Dutch food composition database (NEVO online version 2019/6.0) was used to determine nutritional composition (via energy content (kcal), carbohydrates (in energy percentage (En%)), mono and disaccharides (in En%), fatty acids (in En%), saturated fatty acids (SFA) (in En%), protein (in En%), fibre (in En%) and salt (in g)) [24].

A final questionnaire was specified for the control and experimental conditions. First, a set of general questions was provided, covering participants’ sex, height and weight, educational level, income, living situation, and, for instance, frequency of meat consumption. Depending on the experimental conditions, the questionnaire contained (three or six) additional questions. Participants in the Information nudge condition and Price condition were asked three additional questions as to whether the notification (on the information nudge or price increase, respectively) before entering the virtual supermarket had been read (Yes or No), understood (Yes or No) and whether it had influenced the shopping behaviour (7-point Likert scale: 1 “not at all” to 7 “extremely”). In the Combination condition, the questions on the information nudge as well as on the higher meat prices were asked. Finally, the questionnaire covered questions about participants’ understanding of the software (5-point Likert scale: 1 “strongly disagree” to 5 “strongly agree”), whether the participants’ virtual supermarket groceries corresponded with their usual groceries (5-point Likert scale: 1 “strongly disagree” to 5 “strongly agree”), and whether the participants’ shopping budget was more, the same or less than usual. Also, participants’ attention to the prices and the influence of pricing on their purchases were measured (7-point Likert scale: 1 “not at all” to 7 “extremely”).

### Statistical analysis

Characteristics of the population and secondary outcomes were summarised with descriptive statistics in means and standard deviations (SD) or median and interquartile range (IQR) for continuous variables, and in numbers and percentages for categorical variables. Outcomes were visually inspected for normality using Q,Q-plots and Kolmogorov -Smirnov tests. The primary measure total amount of meat purchases followed a normal distribution. Linear regression models with the total amount of meat purchases as a dependent variable and the conditions as independent variables were used to examine the potential effect modifier education level, as individuals with a lower socio-economic position might respond differently upon the interventions [13]. The variable educational level was added to the unadjusted model with interaction terms between the variable and the intervention conditions to examine effect modification. Interaction terms were not statistically significant (*p* > 0·05) and therefore removed from the model. In the first model, the variable household size was added to the model since this variable is a strong predictor for the total amount of (meat) purchases (model 1). Certain imbalances in characteristics were observed between the conditions, although the drop-out across study conditions was similar. In the second model, further adjustments were therefore made for sex, BMI and educational level to correct for imbalances between the conditions (model 2). Parameter estimates were obtained using generalised linear models and included regression coefficients (β) (representing the absolute mean difference in meat purchases (in g per household per week) for the experimental conditions relative to the control condition (reference) and 95% confidence interval (95%CI) of the mean difference. A sensitivity analysis was performed, in which participants in the experimental conditions were excluded who did not read or understand the notifications before entering the supermarket. Furthermore, in a second sensitivity analysis participants were excluded who defined themselves as vegan, vegetarian or pescatarian. Participants were excluded for analysis if fewer than or equal to five different products were purchased since this type of grocery shopping is not representative of a typical weekly shop. The statistical analysis was performed using SAS software, version 9.4 (SAS Institute Inc., Cary, NC, USA). A two-sided *p*-value of < 0·05 was considered statistically significant.

### Role of the funding source

The funder of the study had no role in study design, data collection, data analysis, data interpretation, or writing of the report.

## Results

**Fig. 1:**
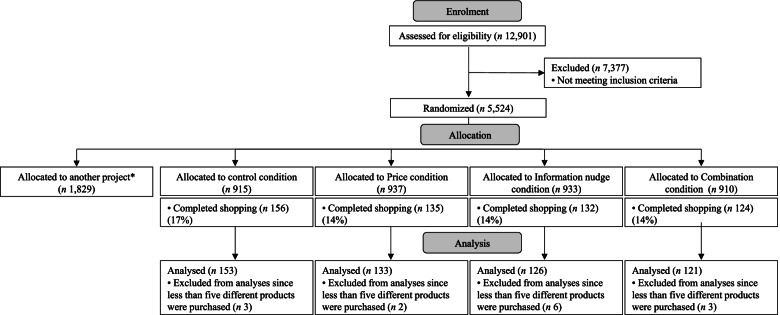
Flowchart of enrolment and allocation of the study participants. *1829 participants were randomised for the purpose of another project (Netherlands Trial Register registration number NL8616)

From 22 June 2020 to 28 August 2020, 150,514 panel members were invited to participate in the study. Of these, 12,901 individuals completed the screening questionnaire (Fig. 1). A total of 5524 participants were eligible for inclusion and randomised and allocated to the control and experimental conditions (*n* = 3695) or to the research conditions of another project (*n* = 1829). (Netherlands Trial Register registration number NL8616). Overall, 547 participants were able to complete the virtual shopping (15%). Participants who completed the shopping were on average younger (mean =  48·3, SD= 16·2 y) compared with those who dropped out of the study (mean = 57·4,  SD = 15·7 y) (Supplemental Table 2). Moreover, participants included in the study were more often higher educated (50%) compared to those who dropped out of the study (29%). After excluding non-representative shops (*n* = 14), the final sample for analysis included 533 participants (*n* = 153 for the ‘Control condition’, *n* = 133 for the ‘Price condition’, *n* = 126 for the ‘Information nudge condition’ and *n* = 121 for the ‘Combination condition’). Characteristics of participants are presented in Table 1. Descriptive statistics show that 9·8% of the participants in the ‘Control condition’ did not purchase meat items in the virtual supermarket. In the ‘Price condition’, ‘Information nudge condition' and ‘Combination condition’, 12·0, 9·5 and 15·7% of participants did not purchase meat products, respectively.

**Table 1:** Population characteristics for control and experimental conditions

	Total population (***n*** = 533)		Control condition (***n*** = 153)		Price condition (***n*** = 133)		Information nudge condition (***n*** = 126)		Combination condition (***n*** = 121)	
**Sex**										
Male	253	47·5%	75	49·0%	67	50·4%	58	46·0%	53	43·8%
Female	278	52·2%	78	51·0%	65	48·9%	67	53·2%	68	56·2%
Other	2	0·4%	0	0·0%	1	0·8%	1	0·8%	0	0·0%
**Age (years)**	48·3	16·2	48·6	16·3	48·4	15·9	46·9	16·7	49·3	15·8
**Household size**	2·3	1·2	2·3	1·3	2·2	1·1	2·4	1·3	2·4	1·2
*% persons > 13 y*	29·1	28·4	28·5	26·8	29·7	29·7	28·4	28·7	29·9	29·0
**Educational level**										
Low	83	15·6%	20	13·1%	23	17·3%	23	18·3%	17	14·0%
Moderate	180	33·8%	45	29·4%	41	30·8%	42	33·3%	52	43·0%
High	270	50·7%	88	57·5%	69	51·9%	61	48·4%	52	43·0%
**BMI (kg/m**_**2**_**)**	26·5	5·8	27·5	6·0	25·9	5·5	26·7	5·9	25·6	5·5
**Weight status**^a^										
Normal weight < 25	243	45·6%	65	42·5%	64	48·1%	58	46·0%	56	46·3%
Overweight (≥25–30)	171	32·1%	50	32·7%	39	29·3%	38	30·2%	44	36·4%
Obese (> 30)	107	20·1%	37	24·2%	25	18·8%	28	22·2%	17	14·0%
**Meat consumption (frequency/week**)										
0	34	6·4%	9	5·9%	8	6·0%	6	4·8%	11	9·1%
< 1	8	1·5%	3	2·0%	2	1·5%	1	0·8%	2	1·7%
1–2	71	13·3%	22	14·4%	18	13·8%	15	11·9%	16	13·2%
3–4	179	33·6%	38	24·8%	51	38·3%	42	33·3%	48	49·7%
5–7	241	45·2%	81	52·9%	54	40·6%	62	49·2%	44	36·4%
**Type of (meat) consumer**										
Vegan	7	1·3%	0	0·0%	4	3·0%	1	0·8%	2	1·7%
Vegetarian	20	3·8%	8	5·2%	3	2·3%	4	3·2%	5	4·1%
Pescatarian	5	0·9%	1	0·7%	1	0·8%	1	0·8%	2	1·7%
Flexitarian	20	35·3%	48	31·4%	46	34·6%	45	35·7%	49	40·5%
Meat consumer	313	58·7%	96	62·7%	79	59·4%	75	595·%	63	52·1%
**Purchased meat**										
**Yes**	471	88·4%	138	90·2%	117	88·0%	114	90·5%	102	84·3%
**No**	62	11·6%	15	9·8%	16	12·0%	12	9·5%	19	15·7%
**Grocery responsibility**										
Entirely	337	63·2%	99	64·7%	87	65·4%	78	61·9%	73	60·3%
Largely	196	36·8%	54	35·3%	46	34·6%	48	38·1%	48	49·7%
**Household monthly income (gross in €)**										
Low (0–2000)	136	25·5%	38	24·8%	34	25·6%	26	20·6%	38	31·4%
Moderate (2000–3000)	135	25·3%	37	24·2%	41	30·8%	33	26·2%	24	19·8%
High (3000+)	262	49·2%	78	51·0%	58	43·6%	67	53·2%	59	48·8%
**Household weekly food expenditures (in €)**										
0–59	170	31·9%	52	34·0%	42	31·6%	40	31·7%	36	29·8%
60–99	205	38·5%	55	35·9%	49	36·8%	53	42·1%	48	39·7%
≥ 100	158	39·6%	46	30·1%	42	31·6%	33	26·2%	37	30·6%
**Changed purchases due to COVID-19**										
No	442	82·9%	124	81·0%	112	84·2%	99	789·6%	107	88·4%
Yes	91	17·1%	29	19·0%	21	156·8%	27	21·4%	14	11·6%
**Shopping budget in virtual supermarket (in €)**	87·10	31·69	87·11	34·37	85·05	30·28	89·33	34·52	87·02	26·34
% of budget spent	83·4	21·7	84·2	19·8	84·6	21·4	83·4	22·3	81·3	23·6
**Total expenditure (€)**	71·24	28·23	72·28	30·40	70·29	26·37	72·58	27·78	69·58	28·06
**Appreciation of shopping budget**										
More than usual	165	31·0%	49	32·0%	32	24·1%	47	37·3%	37	30·6%
Same as usual	256	48·0%	71	46·2%	67	50·4%	62	49·2%	56	46·3%
Less than usual	112	21·0%	33	21·6%	34	25·6%	17	13·5%	28	23·1%
**Price awareness**^**b**^	4·0	1·6	3·9	1·6	4·1	1·6	3·8	1·7	4·1	1·6
**Understanding virtual supermarket**^**c**^	4·6	0·6	4·5	0·6	4·6	0·6	4·5	0·6	4·6	0·7
**Comparability to real-life purchases**^**d**^	4·1	0·8	4·0	0·8	4·1	0·8	4·1	0·8	4·0	0·8

Data are n (%) or mean (SD). *BMI* Body Mass Index

^a^12 missing values

^b^Measured by one item “The program was easy to understand” indicated on a five-point Likert scale ranging from 1 “strongly disagree” to 5 “strongly agree”

^c^Measured by two items “To what extent did you notice prices in the virtual supermarket?” and “To what extent did prices influence your choices in the virtual supermarket?” indicated on a seven-point Likert scale ranging from 1 “not at all” to 7 “extremely”

^d^Measured by one item “The products I have purchased in the virtual supermarket are comparable to my regular food purchases in real-life” indicated on a five-point Likert scale ranging from 1 “strongly agree” to 5 “strongly agree”

**Fig. 2:**
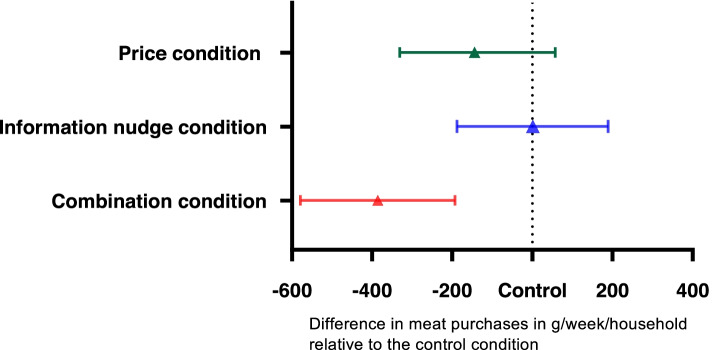
Mean difference in meat purchases in gram per household per week for the experimental conditions compared with the control condition. Estimates are derived from linear regression models adjusted for sex, BMI and educational level. The reference indicates the control condition. Error bars indicate 95% confidence intervals

In linear regression analysis adjusted for household size, − 367 g (95%CI: − 557, − 178) meat per household per week was purchased in the ‘Combination condition’ compared with the ‘Control condition’ (model 1) (Table 2). After further adjustments for sex, BMI and education (model 2), the effect remained significant at − 386 g (95% CI: − 579, − 193) meat purchased in the ‘Combination condition’ compared with the ‘Control condition’ (Table 2; Fig. 2). In the ‘Price condition’, less meat (− 144 g (95%CI: − 331, 43)) was purchased compared with the ‘Control condition’, although not statistically significant. In the ‘Information nudge condition’, the amount of meat purchased was similar to the ‘Control condition’ (1 g (95%CI: − 188, 189)). A sensitivity analysis that excluded participants who did not read or understood the notifications before entering the supermarket did not alter the obtained results (Supplementary Table 3 and 4). Furthermore, a sensitivity analysis in which participants were excluded who defined themselves as vegan, vegetarian or pescatarian resulted in a more pronounced difference in meat purchases in the ‘Combination condition’ compared to the ‘Control condition’ with − 413 g (95%CI − 606, −219) (Supplementary Table 5).

**Table 2:** Effects of price, information nudge and combination condition on total meat purchases in gram per household per week in the virtual supermarket using linear regression analyses

	Price condition (***n*** = 133)			Information nudge condition (***n*** = 126)			Combination condition (***n*** = 121)		
Model 1	− 162	− 347	23	−10	− 198	178	−367	**−557**	**−178**
Model 2	−144	−331	43	1	−188	189	−386	**−579**	**−193**

Data are regression coefficients (β) with 95% confidence intervals. The adjusted amount of meat purchases per household per week and per person per day based on the conditions’ average household size were, respectively, 1084 and 67 g in the control condition, 940 and 61 g in the ‘price condition’, 1085 and 65 g in the ‘Information nudge condition’ and 698 and 4 g 2 in the ‘Combination condition’

Model 1 = adjusted for household size;

Model 2 = model 1 + adjusted for gender (male, female, other), BMI (continuous), education (low, moderate, high)

β represents average difference in gram per household per week compared with the control condition

The mean number of meat items purchased was 4·3  (SD = 3·1) in the ‘Control condition’, 3·7 (﻿SD = 2·4) in the ‘Price condition’, 4·2 ﻿SD = (2·8) in the ‘Information nudge condition’ and 3﻿·2 (﻿SD = 2﻿·4) in the ‘Combination condition’(Table 3). Overall, the purchased food items represented 62·3 (﻿SD =  29·3) kg CO_2_-eq in the ‘Control condition’ and, respectively, 56·3 ﻿(SD =  24·3) and 54·4 (﻿SD =  25·6) kg CO_2_-eq in the ‘Price condition’ and ‘Combination condition’. Shopping baskets in control and experimental conditions contained 30,000–33,000 kcal on average and approximately 13 En% protein, 47–48 En% carbohydrates, 35–36 En% fatty acids, 2 En% Fibre and 74–89 g salt.

**Table 3:** Descriptive statistics for total food purchases, and environmental and nutritional outcomes per household per week for the total population and the control and experimental conditions in the virtual supermarket

	Total population (***n*** = 533)		Control condition (***n*** = 153)		Price condition (***n*** = 133)		Information nudge condition (***n*** = 126)		Combination condition (***n*** = 121)	
**Purchases**										
Purchased food items (n)	39·7	16·4	40·5	15·5	37·6	14·7	41·8	17·3	38·6	15·7
*of which meat (n)*	3·6	2·8	4·3	3·1	3·7	2·4	4·2	2·8	3·2	2·4
**Environmental impact**										
Greenhouse gas emission (kg CO_2_-eq)	58·9	26·8	62·3	29·3	56·3	24·3	61·9	26·6	54·4	25·6
Land use (m^2^/year)	38·3	19·6	39·6	21·4	37·1	18·5	40·1	17·5	36·2	20·2
Blue water consumption (m^3^)	1·70	1·07	1·72	1·02	1·59	1·01	1·76	1·05	1·74	1·21
**Nutritional outcomes**										
Energy (kcal)	32,054	16,164	33,043	18,007	30,710	14,050	33,750	16,072	30,517	15,896
Protein, En%	12·8	3·6	12·7	3·5	12·8	3·4	13·0	3·8	12·8	3·9
Carbohydrates, En%	47·5	10·3	47·7	10·7	47·1	10·8	46·9	9·6	48·2	10·0
Mono- and disaccharides, En%	20·9	8·0	21·8	8·7	20·2	7·8	20·3	7·5	20·9	7·8
Fatty acids, En%	35·7	11·6	35·6	11·0	35·9	11·9	36·3	11·1	35·0	12·3
Saturated fatty acids, En%	11·6	3·7	11·7	3·3	11·5	4·2	11·7	3·5	11·2	3·8
Fibre, En%	2·3	0·8	2·2	0·8	2·3	0·8	2·3	0·8	2·5	0·9
Salt (g) (median (IQR))	81﻿·2	65﻿·4	82·9	64﻿·8	79﻿·5	60﻿·0	89﻿·0	64·9	73﻿·9	66﻿·2

Data are n(%), mean (SD) or median (IQR). *CO*_*2*_*-eq* CO_2_ equivalents, *En*% Energy percentage

## Discussion

Results from this RCT showed that a 30% higher meat price combined with an information nudge on the environmental impact of meat production and consumers role in that regard results in a decrease of − 386 g (95%CI: − 579, − 193) meat per household per week in a virtual supermarket. With the singular fiscal measure of 30% higher meat prices less meat was purchased (− 144 g (95%CI: − 331, 43)). Although the difference was not significant, the reduction was in the expected direction. The singular information nudge did not lead to a change in meat purchases. This study demonstrated the beneficial effects of a higher meat price combined with providing information in order to nudge consumers towards lower meat purchases, which has important implications for planetary and public health.

Lately, more literature has become available on the modelled effects of a meat tax [5, 8, 9] and on behaviour oriented studies that investigate willingness or intentions to reduce meat purchases or consumption [16, 17]. Studies that investigate actual reductions in meat purchases or consumption are scarce. To the best of our knowledge, this is the first study that investigated the effect on meat purchases of different policy measures in a supermarket setting. The mixed policy including both the price increase and the information nudge was effective in reducing meat purchases. In line with the literature, singular or informative measures are often less effective in achieving dietary change compared with more robust measures such as fiscal measures or mixed policies with more pronounced effects [7, 28].

One study was identified that examined the effect on meat meals of higher meat prices in a real-life setting. In the experiment of Garnett et al. (2021), the effect on meal choices of altering the prices of meals with or without meat was studied in a university cafeteria in the UK [18]. The difference between the price of meals with or without meat was 8 %, corresponding to £0·40 (€0·46). In contrast, the price increase in our study was 30% or on average €0·96 per meat item as sold. Similar to the present study, participants were aware of the price increase. During the study period, the price changes were advertised (e.g. on screens on campus, on the menus). The advertisement stated that the prices of meals were changing to reflect the cost of ingredients. Although sales of vegetarian meals increased by 3·2%, the sales of meat meals did not change compared with the baseline [18]. In the study of Garnett et al. only a fiscal measure was studied; we also included an information nudge. This might explain why we observed a significant reduction in meat purchases.

In our study, the singular measure of 30% higher meat prices led to the expected result of less meat purchases, although not statistically significant compared with the ‘Control condition’. This result is more in line with the experiment of Garnett et al. [13] In general, taxing unhealthy foods to discourage their consumption, with or without other intervention components, are effective measures in improving diets and healthy behaviour [13, 14]. Modelling studies have demonstrated the effectiveness of meat taxes previously; however, they did not include an information nudge in their modelling strategy [5, 8, 9, 16]. Our results suggest that including an information nudge may enforce the effect of a fiscal measure.

With the ‘Information nudge condition’, we did not observe any difference in meat purchases compared with the ‘Control condition’. Previous systematic reviews investigating experiments that targeted changing attitudes demonstrated that providing information was successful in changing the intention or willingness to reduce meat purchases or consumption. However, actual reductions in meat purchases or consumption were not observed or measured [16, 17]. In a meta-analysis of experiments, information nudges (framed as cognitive nudges) were found to be the least effective type of nudges in affecting selection and consumption outcomes [29]. In contrast, Harbers et al. (2020) examined the effect of information nudges (providing information on the foods at the point of choice in real-life supermarkets or messages via posters in cafeterias) in real-life food purchase environments. The effects of those information nudges on purchases were heterogeneous but showed modest benefits [11]. The information nudge in the current study was provided shortly before entering the supermarket and not at the point of choice or for a longer period of time nor more frequently exposed, which might be a reason for our contradictory results.

Significant strengths of this RCT include the design and the empirical evidence on consumer changes in meat purchases as a result of a price increase, simultaneously with the information nudge in a virtual supermarket. Previous studies often focused solely on behavioural factors such as willingness or intention to purchase or consume less meat and often relied on self-reported measures [16, 17].

Some limitations should be noted when interpreting our study results. Firstly, the experiment was conducted in a virtual supermarket, which has its limitations. The virtual supermarket has a smaller grocery offer compared to a real-life supermarket. Moreover, participants’ shopping behaviour might be influenced in the virtual supermarket as participants did not spend their own money and they did not receive the groceries. Nevertheless, the New Zealand version of the virtual supermarket, which uses the same methodology as the Dutch version, is previously validated. The validation study compared within persons the real-life groceries with shopping patterns in the virtual supermarket and showed that purchased foods in the virtual supermarket were a good reflection or representation of purchases in a real supermarket [21]. Furthermore, in our study, participants reported that they mostly agreed that their shop reflected their usual groceries (mean score of 4·05 on a 5-point Likert scale). Secondly, although the research team had conducted several steps to minimize drop out, there was a large but equal drop-out of study participants across the study conditions after randomisation. This might have implications for the interpretation of the study results and their external validity. Participants who dropped out of the study were on average older and often had a lower educational level. Since elderly or those with a lower educational level are often less computer literate, this might explain the higher drop-outs among those older or lower educated participants [30]. Despite the drop-out across study conditions was similar, certain imbalances in characteristics between the conditions were observed. To correct for those imbalances we have adjusted the models for the imbalanced variables (sex, BMI and educational level). Moreover, in general selection bias occurs in trials. To minimize selection bias, participants were recruited via a large online research panel with more than 100,000 members, not aware of study aims and were randomised to the control and experimental conditions. Furthermore, the recruitment of participants and study execution was partly during the lockdown in the COVID-19 pandemic. During the recruitment period there were certain restrictions for grocery shopping in the Netherlands. We expect that the COVID-19 pandemic did not have a major influence on our study outcomes since 82·9% of the participants reported that their food purchases were not changed due to the COVID-19 pandemic. The potential effects of a reduction of meat consumption on human and planetary health could be significant. For instance, a Swedish modelling study with an environmental tax of 8·9% to 33·3% on three meat products (beef, pork and chicken) and four dairy products showed 12% lower GHG emissions from the livestock sector [8]. Furthermore, the recent SCBA from the Netherlands that modelled and monetised the 30-year societal effects of 30% higher meat prices demonstrated that daily meat consumption would decrease by 17 g or 16% [5]. As a result, 5550–29,398 cases for diabetes type 2 prevalence would be averted, 2122–6691 QALYs were gained, and the environmental impact (assessed via GHG emissions, acidification, eutrophication of marine and fresh water, and water and land use) decreased by 16%. Overall, this resulted in benefits of between €4100 - €12,300 million over 30 years [5]. In comparison, we found a 23-g reduction per person per day based on the conditions’ average household size of 2·4 persons and a decrease of 386 g meat per week. Furthermore, when taking into account the average adjusted meat purchases in the ‘Control condition’ and ‘Combination condition’ with 1084 g and 698 g, respectively, this can be translated into a relative decrease of 36% in meat purchases per household per week. Therefore, it can be expected that the impact on human and planetary health would be even more significant compared with modelling results from Broeks and colleagues [5]. Future large-scale research is needed to confirm our results in real-life supermarkets.

In conclusion, achieving the most pronounced effects on reductions in meat purchases requires a policy mixture of pricing and informational nudging. This study demonstrated that a 30% price increase for meat is effective in decreasing meat purchases when combined with an information nudge on the environmental impact of meat production and the consumers’ role in that regard. The results could be used to design evidence-based policy measures to reduce meat purchases.

## Supplementary Information

**Additional file 1.**

## Data Availability

The datasets used during the current study are available from the corresponding author on reasonable request.
