# Publisher Correction: Less meat in the shopping basket: the effect on meat purchases of higher prices, an information nudge and the combination: a randomised controlled trial

**DOI:** 10.1186/s12889-022-13601-2

**Published:** 2022-07-19

**Authors:** R. E. Vellinga, M. Eykelenboom, M. R. Olthof, I. H. M. Steenhuis, R. de Jonge, E. H. M. Temme

**Affiliations:** 1 National Institute for Public Health and the Environment, Antonie van Leeuwenhoeklaan 9, Bilthoven, 3721, MA The Netherlands; 2 Department of Health Sciences, Faculty of Science, Vrije Universiteit Amsterdam, Amsterdam Public Health Research Institute, De Boelelaan 1085, 1081 HV Amsterdam, The Netherlands

**Correction: BMC Public Health 22, 1137 (2022)**

**https://doi.org/10.1186/s12889-022-13535-9**

During the publication process of the original article [1] several corrections were not implemented. The correct and incorrect information is shown in this correction article, the original article has been updated with the correct information. The publisher apologizes to the authors & readers for the inconvenience caused.

**Incorrect**

- Achieving the most pronounced effects on reduced meat purchases will require a policy mixture of pricing and informational nudging
- Table 1: 595・%
- Table 1: 789・6%
- Table 1: 156・8%
- Result section: 4・2 SD = (2・8)

**Correct**

- Achieving the most pronounced effects on reduced meat purchases will require a policy mixture of pricing and an information nudge
- Table 1: 59・5%
- Table 1: 78・6%
- Table 1: 15・8%
- Result section: 4・2 (SD = 2・8)
