# Supplementary material for: Less meat in the shopping basket. The effect on meat purchases of higher prices, an information nudge and the combination: a randomised controlled trial
Source: BMC Public Health. 2022 Jun 7;22:1137. doi: 10.1186/s12889-022-13535-9 (PMC9171470; doi:10.1186/s12889-022-13535-9)
Supplement: Supplementary file 1 — Additional file 1. [file 12889_2022_13535_MOESM1_ESM.docx]

SUPPLEMENTAL TABLES

Supplemental Table 1. Overview of meat products being sold in the Virtual Supermarket with unit and price for control and experimental conditions.

Supplemental Table 2. Supplemental Table 2. Characteristics of participants randomized, dropped out and included in the study.

Supplemental Table 3. Overview of participants in the experimental condition that have read and understand the information nudge that they were exposed to in the conditions.

Supplemental Table 4. Sensitivity analysis excluding participants who did not read the information nudge or notification on price increase. Effects of the price condition, information nudge condition and condition on total meat purchases (in gram) using linear regression analyses.

Supplemental Table 5. Sensitivity analysis excluding participants who reported themselves as vegan, vegetarian or pescatarian. Effects of the price condition, information nudge condition and condition on total meat purchases (in gram) using linear regression analyses.

| Supplemental Table 1. Overview of meat products being sold in the Virtual Supermarket with unit and price for control and experimental conditions. | | | | | |
| --- | --- | --- | --- | --- | --- |
| Product | Quantity as being sold | | Regular price (€/unit) | 30% price increase (€/unit) | Product group |
| Chickenwings (frozen) | 750 g |  | 2,55 | 3,32 | Poultry |
| Turkey fillet | 250 g |  | 3,25 | 4,23 | Poultry |
| Chicken drumsticks | 600 g |  | 2,45 | 3,19 | Poultry |
| Chicken burger | 205 g | (2 pieces) | 1,73 | 2,25 | Poultry |
| Chicken breast | 300 g | (2 pieces) | 3,25 | 4,23 | Poultry |
| Bratwurst | 240 g | (2 pieces) | 2,49 | 3,24 | Pork |
| Gelderland smoked sausage | 200 g |  | 2,04 | 2,65 | Pork |
| Frankfurters (can) | 400 g |  | 1,62 | 2,11 | Pork |
| Lean smoked sausage | 200 g |  | 2,00 | 2,61 | Pork |
| Lean bacon strips | 300 g |  | 2,69 | 3,50 | Pork |
| Shoulder chop (pork) | 360 g |  | 2,78 | 3,61 | Pork |
| Spareribs (frozen) | 750 g |  | 5,79 | 7,53 | Pork |
| Pork fillet slices | 260 g | (2 pieces) | 3,04 | 3,95 | Pork |
| Steak (fresh) | 275 g |  | 5,14 | 6,68 | Beef |
| Roast beef | 120 g |  | 3,12 | 4,06 | Beef |
| Hamburger (fresh) | 400 g | (4 pieces) | 2,99 | 3,89 | Beef |
| Hamburgers (frozen) | 840 g |  | 2,52 | 3,28 | Beef |
| Ground beef (fresh) | 300 g |  | 2,29 | 2,98 | Beef |
| Beef tartare (fresh) | 200 g | (2 pieces) | 2,19 | 2,85 | Beef |
| Unox Hamburgers | 160 g |  | 2,39 | 3,11 | Beef |
| Frikadellen (frozen) | 340 g | (4 pieces) | 1,48 | 1,92 | Miscellaneous |
| Frikandellen (frozen) | 1360 g | (16 pieces) | 3,59 | 4,66 | Miscellaneous |
| Chopped half and half | 300 g |  | 1,99 | 2,59 | Miscellaneous |
| Venison steak | 250 g |  | 9,25 | 12,03 | Miscellaneous |
| Lamb chop | 230 g | (4 pieces) | 7,42 | 9,65 | Miscellaneous |
| Ham (slices) | 250 g |  | 3,59 | 4,67 | Cold meat cuts |
| Farmer's sausage | 190 g |  | 3,49 | 4,54 | Cold meat cuts |
| Sandwich sausage (slices) | 150 g |  | 1,99 | 2,59 | Cold meat cuts |
| Carpaccio | 123 g |  | 2,69 | 3,50 | Cold meat cuts |
| Cervelate (slices) | 105 g |  | 2,01 | 2,61 | Cold meat cuts |
| Chorizo | 250 g |  | 2,53 | 3,29 | Cold meat cuts |
| Fillet American | 160 g |  | 2,49 | 3,24 | Cold meat cuts |
| Roast chicken fillet (slices) | 125 g |  | 2,80 | 3,64 | Cold meat cuts |
| Guelderian boiled sausage | 250 g |  | 2,09 | 2,72 | Cold meat cuts |
| Gold salami (slices) | 125 g |  | 2,49 | 3,24 | Cold meat cuts |
| Liver pate (canned) | 168 g | 3 x 56 g | 1,29 | 1,68 | Cold meat cuts |
| Liverwurst | 500 g |  | 2,83 | 3,68 | Cold meat cuts |
| Breakfast bacon (smoked) | 150 g |  | 2,89 | 3,76 | Cold meat cuts |
| Ox sausage | 200 g |  | 2,60 | 3,38 | Cold meat cuts |
| Pate | 170 g |  | 2,24 | 2,91 | Cold meat cuts |
| Salami (sausage) | 250 g |  | 1,51 | 1,96 | Cold meat cuts |
| Shoulder ham (slices) | 150 g |  | 2,29 | 2,98 | Cold meat cuts |
| Smac (can) | 250 g |  | 2,29 | 2,98 | Cold meat cuts |
| Sliced ​​sausage | 105 g |  | 2,01 | 2,61 | Cold meat cuts |

| Supplemental Table 2. Characteristics of participants randomized, dropped out and included in the study. | | | | | | |
| --- | --- | --- | --- | --- | --- | --- |
|  | Total randomized  (n=3,695) | | Dropped-out  (n=3,248) | | Included  (n=547) | |
| Age (y) | 56·0 | 16·1 | 57·4 | 15·7 | 48·3 | 16·2 |
| Responsibility grocery | 2425 | 66% | 2076 | 66% | 349 | 64% |
| Totally responsible |  |  |  |  |  |  |
| Largely responsible | 1270 | 34% | 1072 | 34% | 198 | 36% |
| Educational level | | | | | | |
| Low | 1239 | 34% | 1151 | 37% | 88 | 16% |
| Moderate | 1279 | 35% | 1094 | 35% | 185 | 34% |
| High | 1177 | 32% | 903 | 29% | 274 | 50% |
| Data are mean (SD) or n (%). |  |  |  |  |  |  |

| Supplemental Table 3. Overview of participants in the experimental condition that have read and understand the information nudge that they were exposed to in the conditions. | | | | | | |
| --- | --- | --- | --- | --- | --- | --- |
|  | Price condition (n=133) | | Information nudge condition (n=126) | | Combination condition (n=121) | |
| Did you read the following sentence before you entered the virtual supermarket? The government has increased the tax on meat in the virtual supermarket, which led to a price increase of 30% for meat.’ |  |  |  |  |  |  |
| Yes | 122 | 92% |  |  | 115 | 95% |
| No | 11 | 8% |  |  | 6 | 5% |
| Did you understand this message? |  |  |  |  |  |  |
| Yes | 129 | 97% |  |  | 121 | 100% |
| No | 4 | 3% |  |  |  |  |
| To what extent has this message influenced your choice? ^1^ | 2·7 | 1·7 |  |  | 2·9 | 1·9 |
| Did you read the following sentence before you entered the virtual supermarket? 'The government wants to reduce the consumption of meat in the Netherlands because meat production damages the environment. You can help reduce environmental damage by purchasing less meat.’ |  |  |  |  |  |  |
| Yes |  |  | 119 | 94% | 106 | 88% |
| No |  |  | 7 | 6% | 15 | 12% |
| Did you understand this message? |  |  |  |  |  |  |
| Yes |  |  | 126 | 100% | 120 | 99% |
| No |  |  |  |  | 1 | 1% |
| To what extent has this message influenced your choice? |  |  | 2·2 | 1·4 | 2·8 | 1·7 |
| Data are mean (SD) or n (%).  ^1^ measured on a 7- point Likert scale: 1 “not at all” to 7 “extremely”. | | | | | | |

| Supplemental Table 4. Sensitivity analysis excluding participants who did not read the information nudge or notification on price increase. Effects of the price condition, information nudge condition and condition on total meat purchases (in gram) using linear regression analyses. | | | | | | | | | | | | | |
| --- | --- | --- | --- | --- | --- | --- | --- | --- | --- | --- | --- | --- | --- |
|  | Price condition  (n=122) ^1^ | | | | Information nudge condition (n=119) ^2^ | | | | Combination condition (n=106) ^3^ | | | |  |
|  | β |  | 95%CI | | β |  | 95%CI | | β |  | 95%CI | |  |
| Model 1 | -159 |  | -353 | 36 | 16 |  | -178 | 210 | -341 |  | -545 | -138 |  |
| Model 2 | -177 |  | -369 | 14 | -6 |  | -198 | 187 | -331 |  | -530 | -131 |  |
| Model 1 = adjusted for household size (continuous);  Model 2 = model 1 + adjusted for gender (male, female, other), BMI (continuous), education (low, moderate, high);  ^1^ = 11 participants excluded; ^2^ = 7 participants excluded; ^3^ = 15 participants excluded. | | | | | | | | | | | | | |

| Supplemental Table 5. Sensitivity analysis excluding participants who reported themselves as vegan, vegetarian or pescatarian. Effects of the price condition, information nudge condition and condition on total meat purchases (in gram) using linear regression analyses. | | | | | | | | | | | | | |
| --- | --- | --- | --- | --- | --- | --- | --- | --- | --- | --- | --- | --- | --- |
|  | Price condition  (n=122) ^1^ | | | | Information nudge condition (n=119) ^2^ | | | | Combination condition  (n=106) ^3^ | | | |  |
|  | β |  | 95%CI | | β |  | 95%CI | | β |  | 95%CI | |  |
| Model 1 | -198 |  | -401 | 6 | 6 |  | -200 | 212 | -373 |  | -583 | -163 |  |
| Model 2 | -181 |  | -369 | 7 | -35 |  | -225 | 155 | -413 |  | -606 | -219 |  |
| Model 1 = adjusted for household size (continuous);  Model 2 = model 1 + adjusted for gender (male, female, other), BMI (continuous), education (low, moderate, high);  ^1^ = 8 participants excluded; ^2^ = 6 participants excluded; ^3^ = 9 participants excluded. | | | | | | | | | | | | | |
